# Measuring sports injuries on the pitch: a guide to use in practice

**DOI:** 10.1590/bjpt-rbf.2014.0110

**Published:** 2015-09-01

**Authors:** Luiz C. Hespanhol, Saulo D. Barboza, Willem van Mechelen, Evert Verhagen

**Affiliations:** 1 Department of Public & Occupational Health, EMGO+ Institute for Health and Care Research, VU University Medical Center, Amsterdam, The Netherlands

**Keywords:** sports injury, prevalence, incidence, public health surveillance, epidemiological monitoring, costs and cost analysis

## Abstract

Sports participation is a major ally for the promotion of physical activity. However, sports injuries are important adverse effects of sports participation and should be monitored in sports populations. The purpose of this paper is to review the basic concepts of injury monitoring and discuss the implementation of these concepts in practice. The aspects discussed are: (1) sports injury definition; (2) classification of sports injuries; (3) population at risk, prevalence, and incidence; (4) severity measures; (5) economic costs; (6) systems developed to monitor sports injuries; and (7) online technology. Only with reliable monitoring systems applied in a continuous and long-term manner will it be possible to identify the burden of injuries, to identify the possible cases at an early stage, to implement early interventions, and to generate data for sports injury prevention. The implementation of sports injuries monitoring systems in practice is strongly recommended.

## Introduction

The pandemic of physical inactivity is a major public health problem of the 21^st^ century^1^^-^ 3. Physical inactivity was responsible for 6% to 10% of non-communicable diseases in 2008 and it is a leading risk factor for mortality^4^, accounting for 5.3 million deaths in the same year^5^. Initiatives have been proposed worldwide in order to promote physical activity^2^^,^ 6. In Brazil, this is also a matter of concern, since the prevalence of physical inactivity in adults is estimated to be around 40%^7^. One of the largest initiatives to promote physical activity in Brazil is the *Academia da Saúde* (Health Gym) project supported by the Brazilian Ministry of Health^8^^-^ 10. This program is aimed at reducing the barriers to the access of physical activity and to decrease the risk of non-communicable diseases by building 4,000 community gyms^8^^,^ 9.

Sports participation may be part of the solution in promoting an active lifestyle, the benefits of which are well known^11^^-^ 15. However, sports injuries are adverse effects of this practice and may hamper participation in physical activities^16^. In addition, there are substantial costs of sports-related injuries, making these injuries also a societal problem^17^^,^ 18. As sports injuries are a barrier to the promotion of physical activity and result in costs for society, efforts should be made to prevent them. It is well recognized that the first step towards sports injury prevention is the measurement of the health and societal burden of sports injuries^19^. This has been done in research, but it is still a challenge to implement on a broad scale in everyday practice. Continuous monitoring of sports injuries should be implemented in any sport environment, whether individual or team sports. Early identification of injury and availability of evidence-based interventions are the key factors for sports injury prevention and treatment, and only with a reliable and valid injury monitoring system is this possible. The purpose of this paper is, therefore, to review the basic concepts of injury monitoring and to discuss the implementation of these concepts in practice in order to provide a guide for those who want to implement sports injury monitoring systems.

## What is sports injury?

**Figure 1.**
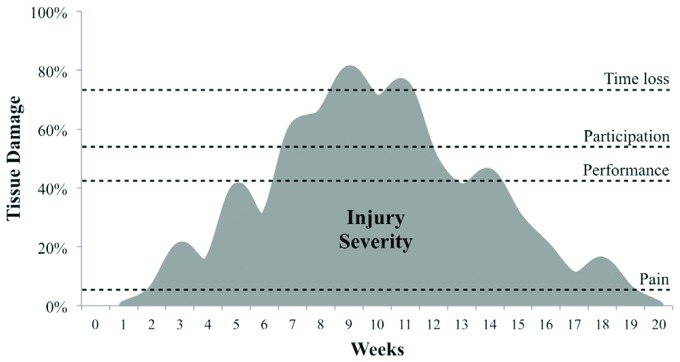
Example of the course of a sports injury over time40. The thresholds (dashed lines) represent the amount of musculoskeletal tissue damage (in percentage) necessary to result in pain, hamper performance, hamper participation in sports, or result in time loss (training sessions or competitions fully missed). The grey area represents the severity or burden related to the injury.

There are many studies addressing the importance of defining 'injury' in research, and this is also an important topic that should be taken into account in practice. In order to truly prevent or manage injuries in the field, firstly it is necessary to define what is considered an injury. Figure 1 exemplifies the course of a musculoskeletal problem (i.e. sports injury) over time. If the definition of injury is based on the symptom "pain", the injury has lasted 17 weeks (week 2 to 19). However, if the definition is based on time loss (i.e. missing training or competition), the injury has lasted 3 weeks (week 8 to 11). In both cases, one is dealing with the same musculoskeletal problem (Figure 1). However, there are two different interpretations. The grey area above the pain or the time loss threshold represents the severity (discussed later in the paper), or the burden caused by the injury, once the definition is based on these thresholds. It is clear that the grey area above the time loss threshold is much smaller than the grey area above the pain threshold, meaning that these two definitions lead to two very different conclusions about the injury severity or burden.

### Sports injury definition

The term 'sports injury' is used to refer to a variety of musculoskeletal damage caused by sports participation^19^. However, 'what is damage?' may be interpreted and recorded in different ways^19^. Recently, studies have provided some 'consensus' helping to standardize the definition and/or classification of injuries^20^^-^ 28, improving the comparability between studies, settings, sports facilities, injury measurement systems, and also between different time-points. There are general definitions, such as 'injuries are considered disorders of the musculoskeletal system or concussions'^28^, and specific definitions, such as injuries requiring medical attention (i.e. any injury that leads to health care utilization) or injuries leading to time loss (i.e. injuries that hamper the ability to fully participate in sports for at least one training session or competition). Also, there are injury definition recommendations for specific sports: cricket^23^, football (soccer)^24^, rugby^25^, tennis^26^, horse racing^27^, athletics^22^, and running^29^. Considering 'what is an injury?' will depend on the specific purpose of the surveillance, which may vary between different sports or settings. However, it is fundamental to appropriately define what is going to be measured^30^.

## Classification

### Mechanism

Different injuries can have different characteristics, causes, and consequences. Therefore, they should be classified in order to elucidate the injury process. The mechanism of the injury drives the initial classification. Acute injuries are those whose onset can be linked to a specific, identifiable and sudden injury event^28^, while overuse injuries are those with a gradual onset mechanism resulting from repetitive micro-trauma, without a specific identifiable event causing the problem^21^. This classification may guide the health care approaches regarding prevention, treatment or prognosis.

### Subsequent injuries

It is not uncommon for an athlete to report more than one injury during a season. Therefore, subsequent injuries should be measured as well. Subsequent injuries can be classified as a new injury (not the same injury as the initial injury, e.g. an injury to another body region) or as a recurrent injury. Recurrent injuries occur in the same body location and usually are of the same nature and/or mechanism. They can be further classified as re-injury (when the injury has fully healed) or as an exacerbation (when the injury has not fully healed)^20^^,^ 31.

### According to consequences

Medical attention and time loss classifications are also very common. They are frequently used to define an injury (as discussed previously). For example, a study involving recreational runners was conducted based on a time loss definition: "[...] any pain of musculoskeletal origin attributed to running and severe enough to prevent the runner from performing at least one training session [...]"^32^. It could also be that the same study had a definition based on medical attention, e.g. "any pain of musculoskeletal origin attributed to running and resulting in a health care professional consultation".

Although using these classifications (i.e. medical attention and time loss) is important to provide information about injuries, using these classifications as injury definitions raises concern. It is possible that athletes do not consult medical professionals for some minor injuries. Additionally, this definition is strictly dependent on medical staff availability, which may not be a reality in many settings. This could result in an underestimation of the number and burden of injuries. Similar reasoning can be used for the application of a time loss definition. Minor injuries are no longer registered or monitored in the injury registration system if they cause no sport time loss (Figure 1).

Minor injuries are not severe in nature; however, they frequently occur in sports and may pose a large problem. In practice, monitoring 'minor' injuries (or complaints) contributes to an early identification of injuries, resulting in the implementation of early interventions to keep these injuries from becoming more severe, lessening the burden on the athlete, team, and/or health care system. Therefore, we suggest using 'medical attention' and 'time loss' concepts as a classification only and not as criteria to define injury.

### Formal and non-formal diagnosis

Injuries are commonly classified according to the body region affected (e.g. ankle) and/or by their nature (e.g. sprain). This helps one to understand which are the most common injuries in a given sport, and therefore guide the prevention and treatment interventions. The best way to do so is to have a formal diagnosis given by a sports health professional or medical staff. However, this is not always possible because of practical/logistic reasons. Therefore, there are other methods to classify such injuries to provide more information about them. Two examples on how to do this in practice are the classifications proposed by Timpka et al.^22^and the Orchard Sports Injury Classification System (OSICS)^33^. In the method of Timpka et al.^22^, an injury can be classified according to body region (e.g. ankle), type of injury (e.g. sprain), and mode of onset (i.e. sudden or gradual). In the OSICS model, an injury is classified with a code containing 4 characters: the first character relates to a body region, the second relates to a specific tissue affected or the pathology, and the third and the fourth characters further describe the pathology or broaden the diagnosis^33^^,^ 34. For example, the code KJAP means Knee injury with a Joint sprain involving the Anterior cruciate ligament, although it is a Partial injury. An isolated rupture would be classified as KJAR.

## Measuring sports injuries

Once the number of injuries is identified, it is time to put this number into context. A number of injuries by itself does not mean much if the number of individuals at risk and/or the sports exposure are not reported. This information will help one to understand the impact/extent of the problem and to make easier comparisons between different time-point measurements in a single population or team, or between different populations or teams. This is important in order to come to conclusions about whether or not the population or team has been reporting more injuries than expected or to be able to generalize the number of injuries to a specific population. Consequently, specific interventions can be discussed and implemented.

### Population at risk and exposure time

Individuals can only be at risk of developing sports injuries if they participate in sports. It does not make sense to measure the proportion of football injuries in individuals who do not play football, for example. Therefore, the population at risk in sports is the population exposed by the sport investigated. Suppose 300 football players were injured during a season. Think about the impact of these 300 injured football players if the source population consisted of 10,000 or 500 football players (i.e. individuals at risk). The probability of having an injury during one season is, in the first case (300/10,000) 0.03, or 3%. In the second case (300/500), the probability is 0.6 or 60%, a much higher figure. Therefore, to measure the burden of injuries, it is necessary to know the total population at risk, or the source population, who have a possibility of being injured.

Exposure time is also a very important measure and concept. Even if all individuals practice sports in a source population (i.e. the population at risk), differences in exposure may lead to differences in injury risk. Individuals who practice sports once a week for one hour (i.e. sports exposure of one hour per week) are less exposed than individuals who practice five days a week for two hours (i.e. sports exposure of 10 hours per week). The practice of sports is a necessary cause for sports injuries^35^. This means that, theoretically, those who are more exposed to the sport activity are more likely to develop a sports injury (if all other variables are controlled). For example, if 50 new injuries were registered in a source population comprised of 200 athletes and the total sports exposure time for this population was 5,000 hours of practice, one could say that the injury risk in this population was 10 injuries per 1,000 hours of practice. However, if the exposure time was 2,000 hours, the injury risk would be 25 injuries per 1,000 hours of practice, which is a risk 2.5 times higher although the number of injuries is the same. Calculations using the entire source population (i.e. population at risk) or the sports exposure are discussed later in the paper.

### Prevalence

Prevalence is the number of people with a given health problem (i.e. the number of cases) in a defined population at any given point in time (Equation 1)^36^. In sports, prevalence is usually reported at a specific point in time (e.g. in the middle of the season) - what is known as 'point prevalence'. However, in some reports, prevalence is also defined as the period prevalence (e.g. entire season). Prevalence is often used to report the overall extent of the sports injury problem. Suppose a sports manager wants to measure how many football players are injured exactly in the middle of a season. It is known that in this specific time-point, 50 out of 500 football players are injured. The prevalence (Equation 1) of football injuries in the middle of the football season could be 0.1 or 10% in this example.



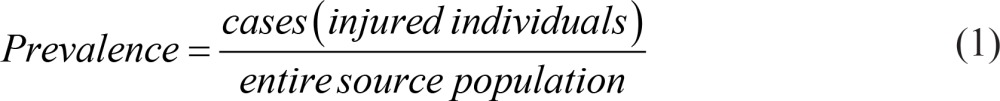



### Incidence

Incidence is the number of new events that occurred in a given population at risk during a period of time^36^. To identify the onset of events (e.g. injuries) and then to be certain that the events are new, a continuous (i.e. longitudinal) measurement is needed. Incidence can be expressed as a proportion (i.e. incidence proportion or risk) by dividing the number of new injured participants (i.e. the number of cases) by the total number of individuals at risk (i.e. the entire source population) during a period of time (Equation 2)^37^. As an athlete may have more than one injury over a period of time (e.g. a season), the clinical incidence can also be calculated. Clinical incidence (Equation 3) is the number of events (i.e. the number of new injuries) divided by the total number of individuals at risk (i.e. the entire source population)^37^.



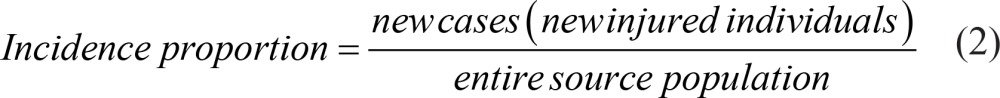





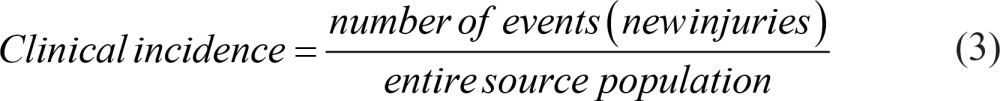



Incidence can also be expressed as incidence density (or incidence rate), i.e. the number of events (NOTE: participants can have more than one injury over a period of time) by the exposure (i.e. person-time) of the sport investigated (Equation 4)^38^. Exposure refers to the period from the beginning to the end of the measurement for non-injured individuals. For injured individuals, the exposure is from the beginning of the measurement until the time the injury was identified (i.e. time-to-injury). Person-time is an epidemiological term often used to describe exposure, and it means that the exposure of each individual was calculated and then added (i.e. the sum of person-time exposure) to the incidence density calculation^37^.

In sports, the exposure can be expressed in such terms as hours of participation, days (training or competition), or km. The incidence density is usually expressed by the number of events per 1,000 or 10,000 person-time exposure. Even though different types of exposure units are described, efforts are needed to achieve a common measure. For instance, a study in field hockey reported an incidence density of 7.87 injuries per 1,000 games, and 3.7 injuries per 1,000 training sessions^39^. Although this information gives the impression that more injuries were identified during games than during training sessions, this conclusion is misleading, because the exposure unit is not the same. A game could have lasted 1.2 hours and a training session could have lasted 5 hours, but they will still count as 1 unit for games and 1 unit for training sessions, making the comparability between the incidence densities problematic. Therefore, the authors suggest that the exposure unit should be expressed using hours of participation in order to facilitate the comprehension and comparison between different sports (e.g. field hockey and football) and types of participation (i.e. training or competition), unless a relevant reason justifies otherwise.



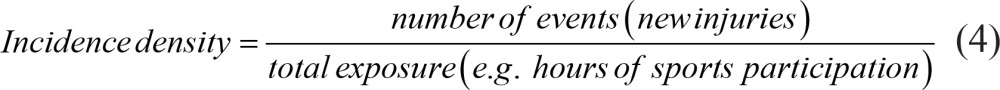



Consider a population of 500 football players. Suppose 70 new injuries were identified in 50 athletes, and the total exposure (i.e. the sum of injured and non-injured person-time exposure) was 20,000 football hours (i.e. both training and competition). The incidence proportion (Equation 2) of this example is 10% (50 new cases divided by 500 individuals at risk) and the incidence density (Equation 4) is 0.0035 (70 new injuries divided by 20,000 hours) or 3.5 injuries per 1,000 hours of sport exposure (0.0035 multiplied by 1,000). Note that the incidence density takes into account the number of injuries, which is suitable since an athlete commonly has more than one injury during a certain period of time (e.g. a season).

### Prevalence and incidence applications

Prevalence rather than incidence is used to describe the overall burden or extent of the sports injury problem. If the question is 'How many athletes are expected to have sports injuries?', the recommended measure would be prevalence. However, most sports managers are more interested in the risk of sports injuries. In this case, incidence proportion is the best option to answer the following question: 'What is the risk of athletes being injured?'. If the athletes can have more than one injury during a period of time and one wants to know 'what the frequency of injuries is in a certain population', then the clinical proportion is a good measure. Incidence density is widely used to answer the following question: 'How many injuries would be expected for a certain amount of exposure?'^37^. This is an interesting question, because an individual cannot have a sports injury if he or she is not exposed to the sport being investigated.

The issue of measuring overuse injuries in sports should also be discussed. By definition, overuse injuries are those injuries with a gradual onset. However, it is very difficult to identify precisely the real onset of these injuries. In addition, the symptoms of an overuse injury could present as a sudden onset, whilst the course of the injury is actually a long-term process. This phenomenon makes things even more difficult^40^. Therefore, it has been suggested that the mean prevalence, calculated based on the time-point prevalences repeatedly measured over time, is a better measure of the sports injury magnitude than incidence from an overuse injury perspective^40^^,^ 41.

## Severity

Measuring injury severity is essential to understand the extent to which sports injuries affect health^19^. Different aspects are used to determine the severity of sports injuries such as: nature of injury, duration, medical attention, sports time loss, working time loss, permanent damage, and costs of sports injuries^42^. This emphasizes the importance of appropriate injury monitoring and classification.

The nature of a sports injury is an indication of its severity. A concussion is more likely to be more severe than a blister. A similar reasoning occurs with the anatomical location of injuries. A blister on the foot or toe of a runner has different consequences than the same injury in a rower. Despite the nature and anatomical location, the extent of symptoms and other consequences of an injury are also crucial. Individual characteristics, the energy involved at the moment of injury occurrence, and the injury mechanism are examples of how the same injury in individuals from the same source population may lead to a different classification of severity.

Mapping the duration of injury also contributes to the measure of severity. For this and other reasons, continuous monitoring (i.e. longitudinal data) is essential. An ankle sprain might be considered more severe than an Achilles tendinopathy in the short term. However, the overuse mechanism of the Achilles tendinopathy might lead to a longer recovery period than an ankle sprain that had an acute mechanism. Therefore, in the long-term, the Achilles tendinopathy may result in greater consequences to the athlete, leading to a higher severity classification than an ankle sprain.

Medical attention and time loss are also examples of severity. An injury that requires medical attention is more severe than an injury that does not. Similarly, if an athlete is not able to participate fully in normal sport activities due to an injury, the time loss indicates the severity of this injury. From a societal perspective, injuries occurring during sports participation may have consequences during other activities. Therefore, working time loss can also be used as a measure of severity, since it is not uncommon that people are not able to work because of a sports injury.

Most athletes recover from sports injuries without a permanent disability (residual symptoms)^42^. However, injuries like concussions with brain damage, spinal injuries, or eye injuries may leave permanent damage. Injuries that cause permanent damage are clearly more severe than injuries that do not. The costs of sports injuries are also important to determine severity, and the discussion about costs can be found in the next section.

## Economic costs

The costs of sports injuries are usually described as a measure of injury severity^42^. In general, a more severe injury leads to higher monetary costs because of such things as medical consultations, medications, medical devices, and productivity loss^42^. All costs related to sports injuries are most commonly taken into account in an economic evaluation, no matter who pays or receives payment^43^. This is a societal perspective approach. There are four typical classifications of economic costs from this perspective^42^^-^ 45:

- Direct costs or health care costs: costs related to health care utilization, such as consultations with a general medical practitioner, sports physician, medical specialist (e.g. orthopedic surgeon), physical therapist, massage therapist, alternative therapist, the use of hospital care, medications, and medical devices (e.g. crutches, tape, braces).
- Indirect costs or lost productivity costs: costs related to loss of productivity due to absenteeism from paid or unpaid work (e.g. household work, loss of study time, loss of leisure time) or due to presenteeism (i.e. not being able to perform fully at work as a result of the injury).
- Societal costs: include insurance administration costs, costs related to insurance programs, workers' compensation costs (i.e. workers may receive wage replacement and/or medical benefits due to sick leave^44)^, and litigation costs (i.e. legal and court costs related to time spent by lawyers and judges, contribution made by legal support services, and overhead expenses).
- Social costs: costs related to the psychological burden of the injury (e.g. depression, social isolation, and economic dependence).

Costs data should be collected and monitored by a reliable and continuous injury registration system^42^. Besides the challenge, the evidence about economic costs of sports injuries has been growing, especially for direct and indirect costs^17^^,^ 18 ^,^ 46 ^,^ 47. Societal and social costs evidence is less common because they are more difficult to measure and estimate. Moreover, social costs are considered "unquantifiable" because of the difficulty in measuring them^42^.

### Challenges in costs data analysis

An economic evaluation requires the collection of data on such things as the number of (para)medical consultations, medications taken, number of medical devices used, loss of paid working productivity (in hours or days), loss of studying hours, and loss of leisure time hours. However, this is not enough. After data collection, it is necessary to transform the number of consultations, loss of productivity, and societal and social consequences into a monetary value.

The Dutch health care system maintains a continuous registration of costs-related data. From a central website^48^, it is possible to download a full report of all the relevant information about the costs related to health care^49^. If additional information is necessary, the Dutch Central Bureau of Statistics website^50^ provides a variety of additional information (e.g. average hours spent during paid work by age and gender^51^). Therefore, the Dutch system allows a very reliable economic evaluation for those who want to perform such analysis in that country.

In Brazil, differences in socioeconomic groups and availability of medical care and costs (e.g. public and private systems) make the economic evaluation even more challenging. However, the Brazilian public health care system (SUS) also keeps continuous records of health-related data through DATASUS^52^. Within this database, it is possible to find a plethora of information such as number of health care consultations and hospitalizations, costs, and per capita income. DATASUS may be an important tool to perform economic evaluations on sports injuries in Brazil and should be used more for this purpose.

## Sports injury monitoring systems

Injury monitoring has been performed in a variety of ways in research and practice. It can vary from very simple and non-validated surveys^32^ to more sophisticated and validated injury management systems^28^^,^ 53. Regardless of the vehicle used to collect the injury data, the aspects discussed previously should be addressed in all of them.

There are several injury monitoring systems that record sports injuries over time in a continuous (i.e. longitudinal) manner and also measure the amount of sports exposure^53-56^. Some of these systems measure exposure indirectly and provide estimations. For example, a team of 50 players with 20 training sessions and 5 competitions may have 1,250 athlete exposures (50 multiplied by 25). Examples of this approach are^56^: National Athletic Injury Reporting System (NAIRS), Canadian Athletic Injury/Illness Reporting System (CAIRS), NCAA Injury Surveillance System, Sports Injury Monitoring System (SIMS), National High School Athletic Injury Registry, and Athletic Injury Monitoring System (AIMS). However, other injury monitoring systems can measure individual sports exposure directly and may be able to provide more accurate measures based on sports exposure (e.g. incidence density). Examples of this approach are^53-56^: Athletic Health Care System (AHCS), Sport Injury/Illness Reporting System (SIIRS), Canadian Intercollegiate Sport Injury Registry, the IOC Injury Surveillance System for Multi-Sports Events, Training and Injury Prevention Platform for Sports (TIPPS), and the Sports Injury Tracker.

There is a debate about differences between systems in measuring acute and overuse injuries^40,41^. Many injuries that occur during tournaments and/or participation in contact sports present an identifiable acute onset, and most of the monitoring systems are effective in identifying these injuries. Because of this, these systems provide reliable information for incidence calculations. However, in many endurance sports, most injuries occur by gradual onset or repetitive movements. The onset and symptoms of overuse injuries are very difficult to record in these systems because they present a gradual and transient mechanism^40^. In this case, incidence is almost impossible to measure accurately. Therefore, a monitoring system was developed in order to deal properly with overuse injuries^41^ and was further broadened to monitor any sort of health problems in sports: the Oslo Sports Trauma Research Center (OSTRC) Questionnaire on Health Problems^28^.

The OSTRC questionnaire^28^ prospectively registers health problems asking 4 key questions: (1) the extent to which injury, illness, or other health problems have affected sports participation; (2) training volume; (3) running performance; and (4) the extent to which the individual has experienced symptoms. Based on the responses, a severity score ranging from 0 to 100 is created. The health problems are further differentiated into illnesses or injuries. For the purposes of this paper, only the sports injury application will be discussed.

The system is based on weekly prevalence measures, and the mean weekly prevalence with its 95% confidence interval (95% CI) has been recommended to be the summary measure. Moreover, it is possible to identify the first report of an injury, and then incidence calculations for acute injuries are also possible. The developers of the questionnaire recommended that medical staff should do the classification of the injuries^28^. However, if this is not possible, the tools previously discussed could be used for this purpose. The severity score provides an overview of the injury course over time and also differentiates periods of lower and higher severity (Figure 1).

Due to the ability of the OSTRC questionnaire to deal with both acute and overuse injuries, our research group has been using this questionnaire to collect injury data on a variety of sports. In addition to the OSTRC questionnaire, sport-specific questions about exposure (usually in hours of training and competition) and costs related to injury are also included. Costs data are usually neglected in injury monitoring systems in spite of their well-recognized importance, and then the overall burden of injuries may be underestimated. The English version of the OSTRC questionnaire can be found elsewhere^28^.

### Example of application and implications for practice

**Figure 2.**
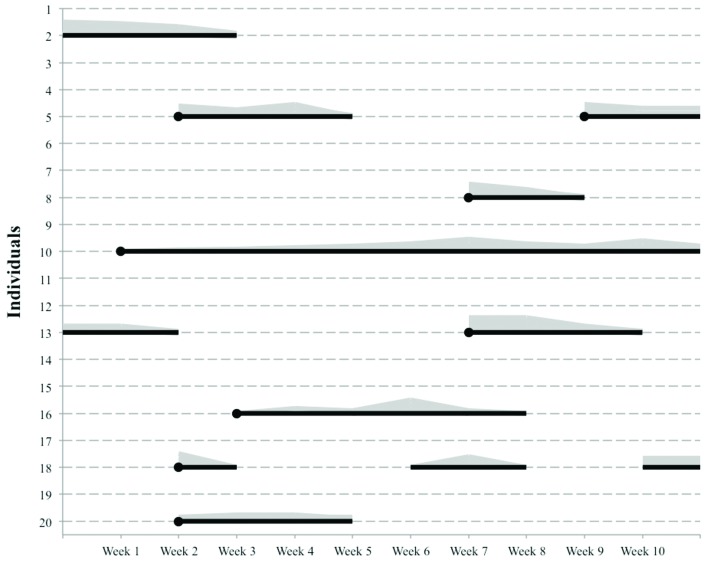
Example of how sports injury monitoring data may be presented in a population level40. The black lines represent the duration of the injuries since their onset or first report (black circles). The grey area represents the variation of severity over time.

An example of how injury data collected by these monitoring systems may be displayed for analysis and interpretation in practice is presented in Figure 2. The black lines represent the duration of the injuries from onset or from the time they are first reported (black circles). The grey area represents the variation in severity over time of each injury in each individual. With this monitoring chart, one can identify the periods when the athletes reported more injuries and/or the severity was worse, for example in weeks 2, 6, and 7 of Figure 2. The implication is that the trainer or the medical staff can analyze what happened during this period (e.g. a specific competition or period in which a specific training program was implemented) and develop a strategy and/or intervention to prevent this from happening again. Moreover, after the action, they can see if the strategy and/or intervention was effective in decreasing the prevalence, incidence, or severity of all or specific injuries while the surveillance is maintained.

A more individual tailored approach could be the early identification of injuries for the implementation of early interventions. This aspect has two implications. Firstly, the early identification and early intervention can prevent a minor injury from becoming a more severe injury with more sports participation, health, and societal consequences. This could be done with the individuals 10 and 16 in Figure 2, because it is clear that in the early stages, the injury severity was not high, but it got worse over time. Maybe this sequence could have been prevented. Secondly, the early identification of an injury leads to an earlier treatment or intervention, which prevents the injury from getting worse and/or avoids permanent damage. Individuals 8 and 20 in Figure 2 are examples of an early identification and intervention leading to a faster recovery.

## Online technology

The usage of online technology is becoming a reality worldwide. It is estimated that more than 40% of the world population used the internet in 2014^57^. In Brazil, more than 50% of the population used the internet in the same year, and this number is growing^58^. Therefore, there is a lot of opportunity to use e-Health, which means "the usage of information and communication technologies (ICT) for health"^59^. In sports, e-Health can be used to monitor injuries in a variety of ways. Online platforms have been used widely in sports injury research, since it is possible to create questionnaires and send a link to these questionnaires (usually by email) and the answers can be downloaded afterward.

Sometimes this requires cooperation between sports, medical, and ICT personnel to create an online platform. However, now there are several commercial online platforms in which one can simply imbed a questionnaire and start using it. Another way to collect data in order to monitor injuries is by text messaging (e.g. short message service: SMS). This method has been increasingly and successfully implemented^60^^-^ 63, since more and more people are using mobile phones or other portable devices (m-Health)^64^.

Advantages of using online platforms include^65^^,^ 66: (1) self-entering data by the participant or athlete eliminating the manual entry by the sports manager, increasing fidelity of the data and decreasing the reporting bias; (2) response fields can be predefined with a reasonable range of possibilities, eliminating errors and out-of-range data; (3) reminders may appear if the individual skips some mandatory questions, eliminating missing data and increasing the accuracy of information; and (4) the possibility of branching questions based on the previous responses, saving time, minimizing the burden of answering the questionnaire, and still maintaining the individuals' motivation to continue answering the questionnaire over time.

Privacy and confidentiality issues are the major concerns about the usage of online technology^64^. Privacy is the right of an individual not to have his/her private information exposed, and confidentiality is the permission to access information by authorized individuals only^67^. An unprecedented amount of an individual's information can be collected and stored in online platforms, and the 'terms and conditions of use' of these platforms cannot violate the privacy and confidentiality rights of the individual. For example, one may have consented to provide information to be used by the team staff, but has not consented for commercial use of the information by third parties^64^. These issues must be considered beforehand to avoid misuse of information.

The use of online technology in sports practice is still challenging. Even with the increasing number of internet and portable device users, not all individuals can be reached by such technology. In addition, different populations may use online resources differently, meaning that the questionnaire should target the population of interest (e.g. adolescents or elderly). Another important aspect is the validity of using existing questionnaires on an online platform. Questionnaires created and tested in a paper version may not have the same clinimetric properties of the online version, thus it should be tested in the online environment. Finally, the online technology does not substitute the personal contact between the athlete and the trainer, medical staff, or sports managers, which is invaluable^64^. It is recommended that both approaches should be used in order to optimize and improve the monitoring of sports injuries^28^^,^ 64.

## Conclusions

Today, the development of a system to monitor injuries in individual or team sports is not only feasible, but also strongly recommended in practice. Many tools have been developed and proven to be implementable and manageable, and they are waiting to be used. This paper reviewed the most important aspects of implementing injury-monitoring systems for sports populations and/or facilities, and we recommend their immediate use. Only with this information collected over the long-term will it be possible to truly identify the burden of injuries; enable early identification of possible cases to prevent them from becoming an injury with greater consequences in sports participation, health and social activities (including work); enable comparisons within or between sports modalities; and providing data for sports injury prevention and intervention. Although plausible considerations may differ between different settings, knowledge provided by continuous injury surveillance in sports practice is the key to the management of sports injuries.
